# Supplementary material for: Resolving the full spectrum of human genome variation using Linked-Reads
Source: Genome Res. 2019 Apr;29(4):635–45. doi: 10.1101/gr.234443.118 (PMC6442396; doi:10.1101/gr.234443.118)
Supplement: Supplemental Material [file supp_gr.234443.118_Supplemental_longranger-2.2.zip › longranger-2.2.tar.bz2!/lr2.2-public/lib/stan/cmdstan/stan/doc/api/html/agrad_2rev_2matrix_2trace__gen__quad__form_8hpp.html]

Stan: src/stan/agrad/rev/matrix/trace\_gen\_quad\_form.hpp File Reference


|  |  |
| --- | --- |
|  | Stan  2.5.0  probability, sampling & optimization |


- Main Page
- Related Pages
- Namespaces
- Classes
- Files

- File List
- File Members

All Classes Namespaces Files Functions Variables Typedefs Enumerations Enumerator Friends Macros Pages

- src
- stan
- agrad
- rev
- matrix

Namespaces |
Functions

trace\_gen\_quad\_form.hpp File Reference

`#include <boost/utility/enable_if.hpp>`  
`#include <boost/type_traits.hpp>`  
`#include <stan/math/matrix/Eigen.hpp>`  
`#include <stan/math/matrix/typedefs.hpp>`  
`#include <stan/agrad/rev/matrix/value_of.hpp>`  
`#include <stan/agrad/rev/var.hpp>`  
`#include <stan/agrad/rev/vari.hpp>`  
`#include <stan/agrad/rev/matrix/typedefs.hpp>`  
`#include <stan/math/matrix/trace_gen_quad_form.hpp>`  
`#include <stan/math/error_handling/matrix/check_multiplicable.hpp>`  
`#include <stan/math/error_handling/matrix/check_square.hpp>`

Go to the source code of this file.

|  |  |
| --- | --- |
| Namespaces | |
|  | stan |
|  | Probability, optimization and sampling library. |
|  | |
|  | stan::agrad |
|  | Function gradients via reverse-mode automatic differentiation. |
|  | |

|  |  |
| --- | --- |
| Functions | |
| template<typename TD , int RD, int CD, typename TA , int RA, int CA, typename TB , int RB, int CB> | |
| boost::enable\_if\_c  < boost::is\_same< TD, var >  ::value\|\|boost::is\_same< TA,   var >::value\|\|boost::is\_same  < TB, var >::value, var >  ::type | stan::agrad::trace\_gen\_quad\_form (const Eigen::Matrix< TD, RD, CD > &D, const Eigen::Matrix< TA, RA, CA > &A, const Eigen::Matrix< TB, RB, CB > &B) |
|  | |

## Variable Documentation

trace\_gen\_quad\_form\_vari\_alloc<TD,RD,CD,TA,RA,CA,TB,RB,CB>\* \_impl

Definition at line 99 of file trace\_gen\_quad\_form.hpp.

Eigen::Matrix<TA,RA,CA> A\_

Definition at line 37 of file trace\_gen\_quad\_form.hpp.

Eigen::Matrix<TB,RB,CB> B\_

Definition at line 38 of file trace\_gen\_quad\_form.hpp.

Eigen::Matrix<TD,RD,CD> D\_

Definition at line 36 of file trace\_gen\_quad\_form.hpp.


---

[ Stan Home Page ]

*© 2011–2014,
Stan Development Team.*
